# Adjuvant Platelet-Rich Fibrin (PRF) Therapy for Wound-Bed Conditioning and Facial Flap Reconstruction: A Systematic Review of Tissue Perfusion and Post-oncological Scar Remodeling

**DOI:** 10.7759/cureus.115109

**Published:** 2026-08-24

**Authors:** Alejandro Gonzalez, Hugo David Miranda de la Paz, Paula Andrea Cuenca Cuevas, Stefany Katherine Correa Villavicencio, Madai Mendoza Herrera, Danna Valentina Díaz Rodríguez, Jessica Daniela de la Rosa Leal, Yamile Itzel Lujano Amparán

**Affiliations:** 1 Clinical Research, Hospital Universitario Mayor, Méderi, Bogotá, COL; 2 Medicine, Universidad del Valle de México, Campus Guadalajara, Guadalajara, MEX; 3 Medicine, Fundación Universitaria Navarra - Uninavarra, Neiva, COL; 4 Medicine, Asociación de Clínica Estética y Reparadora, Córdoba, ECU; 5 Medicine, Universidad Westhill, Ciudad de México, MEX; 6 Medicine, Universidad de Manizales, Ibagué, COL; 7 Internal Medicine, Hospital de Especialidades No. 71 (UMAE 71), Instituto Mexicano del Seguro Social (IMSS), Torreón, MEX; 8 Internal Medicine, Instituto Mexicano del Seguro Social (IMSS), Guadalajara, MEX

**Keywords:** platelet-rich fibrin, reconstructive surgical procedures, skin neoplasms/surgery, surgical flaps, wound healing

## Abstract

Facial oncologic surgery can create defects requiring local, regional, or free-flap reconstruction. Platelet-rich fibrin (PRF) is an autologous fibrin matrix containing platelets and leukocytes that has been investigated as a wound-bed conditioning adjunct; however, its role in post-oncological facial flap reconstruction remains uncertain. The objective of this study is to map literature published from 2022 through 2026 on PRF-assisted wound-bed conditioning relevant to tissue perfusion, flap or graft viability, wound healing, and scar remodeling in facial and head-and-neck reconstruction.

A structured evidence map informed by PRISMA 2020 reporting principles used PubMed/MEDLINE, Scopus, Web of Science, the Cochrane Library, and supplementary reference-list searching. Human clinical, preclinical, and in vitro evidence was grouped separately to avoid conflating evidential weight. Seventeen core reports were included: 16 human clinical reports and one preclinical flap study.

A prospective randomized study of radial forearm flap donor-site reconstruction reported higher skin-graft survival with PRF placed between the wound bed and graft. A case report described delayed deep wound healing after tracheotomy or lymph-node dissection. Adjacent oral and maxillofacial randomized trials reported improvements in selected morbidity or early-healing outcomes, while facial skin studies reported changes in dermal density or scar-related outcomes. To our knowledge, no study directly evaluated objective perfusion, viability, or scar outcomes in post-oncological facial flaps.

PRF is biologically plausible as a wound-bed adjunct, but evidence is insufficient to support routine use in post-oncological facial flap reconstruction. Future multicenter studies should use standardized PRF protocols and objective perfusion and scar measures, including laser Doppler methods, indocyanine green angiography, and validated scar scales.

## Introduction and background

Surgical management of facial cutaneous malignancies is common. Current recommendations for basal cell carcinoma include surgical excision and radiation therapy, both of which can result in disfigurement or functional compromise at critical facial sites [1]. Mohs micrographic surgery maximizes tissue preservation while achieving margin control and can facilitate functional and cosmetic reconstruction [2-3]. Post-Mohs and post-excisional facial reconstruction is more than a closure problem: outcomes depend on patient comorbidities, defect size, anatomic subunit, timing of repair, reconstructive technique, and postoperative complications [2]. Local advancement, rotation, and transposition flaps, together with skin grafts, remain central reconstructive options because they provide tissue with similar color, thickness, and texture. Their outcomes, however, can be limited by compromised vascularity, excessive tension, dead space, infection, radiation injury, and scar contraction [3].

Platelet-rich fibrin (PRF) is a second-generation autologous platelet concentrate embedded in a fibrin matrix. Contemporary reviews describe leukocyte- and platelet-rich fibrin (L-PRF) as a source of platelets, leukocytes, cytokines, fibrin architecture, and growth factors with potential roles in inflammatory modulation, hemostasis, antimicrobial activity, angiogenesis, and soft-tissue regeneration [4]. Clinical guidance also emphasizes that L-PRF products generated with different centrifugation systems are not interchangeable: centrifugation parameters, tube composition, processing time, compression, membrane thickness, and liquid versus solid formulation can alter the resulting biologic product [5]. Oral-surgery literature has expanded the clinical use of PRF while consistently identifying protocol heterogeneity and persistent uncertainty regarding comparative effectiveness [6].

Evidence in dermatology and facial soft tissue is broader but remains indirect. PRF has been investigated in chronic wounds, alopecia, facial rejuvenation, acne scars, and periorbital skin quality, but these indications are not equivalent to post-oncological flap reconstruction [7]. Oncologic safety also requires caution. In vitro work has shown that liquid blood concentrates can alter proliferation and cell-cycle- or apoptosis-related gene expression outcomes in nonmelanoma skin-cancer cells; consequently, placement near an incompletely cleared malignancy cannot be assumed to be safe [8].

A substantial evidence gap therefore remains. The structured review question was: in patients with facial or head-and-neck oncologic defects undergoing flap, graft, or wound-bed reconstruction, does adjunctive PRF, compared with standard reconstruction or no PRF, improve perfusion, vascularization, flap or graft viability, wound healing, or scar outcomes? Most available clinical studies use pain, swelling, epithelialization, graft take, or skin thickness as outcomes, whereas objective measurements of flap perfusion - such as laser Doppler flowmetry, indocyanine green angiography, tissue oxygenation, or human microvascular density - are uncommon. The 2022-2026 evidence window was selected to map contemporary PRF preparations, reconstructive techniques, and outcome measures; it was not intended to establish historical comprehensiveness. The review explicitly distinguishes direct reconstructive evidence from adjacent soft-tissue evidence because extrapolation from oral mucosa, aesthetic skin, or animal flap models would otherwise overstate clinical certainty.

## Review

Methodology

Review Design and Structured Question

This review was conducted as a structured evidence map informed by PRISMA 2020 reporting principles [9]. The population of interest comprised patients with facial or head-and-neck oncologic defects undergoing flap or graft reconstruction and/or wound-bed management. Eligible interventions included PRF in solid membrane, leukocyte-rich, advanced, or injectable formulations. Comparators included standard reconstruction, no PRF, platelet-rich plasma (PRP), hyaluronic acid, photobiomodulation, and conventional wound care. Primary outcomes were tissue perfusion, vascularization, angiogenesis markers, flap or graft survival, necrosis, and viability. Secondary outcomes included wound healing, epithelialization, pain, swelling, infection, dehiscence, patient morbidity, scar quality, skin thickness, dermal density, and cosmetic outcomes.

Information Sources and Search Strategy

Information sources were PubMed/MEDLINE, Scopus, Web of Science, the Cochrane Library, and reference-list searching. The core search string was: ("platelet-rich fibrin" OR PRF OR L-PRF OR A-PRF OR i-PRF OR "leukocyte platelet-rich fibrin") AND (facial OR face OR maxillofacial OR "head and neck" OR Mohs OR oncologic OR cancer OR basal OR squamous) AND (flap OR reconstruction OR "wound bed" OR perfusion OR angiogenesis OR vascularization OR "scar remodeling" OR POSAS OR "Vancouver Scar Scale" OR healing). The evidence window was 2022-2026. English-language human evidence was prioritized; preclinical flap models and in vitro oncologic safety studies were considered only when direct human facial-flap evidence was absent. Database-specific translations and exact search dates were not available in the documented review file. 

Eligibility Criteria and Evidence Grouping

Two eligibility levels were applied. Direct evidence comprised PRF used for wound-bed management in head-and-neck reconstructive surgery following oncologic and/or flap-related procedures. Adjacent-support evidence comprised oral or maxillofacial soft-tissue randomized trials, facial dermatologic remodeling studies, medication-related osteonecrosis of the jaw (MRONJ) or chronic-wound studies, and preclinical flap experiments. Human clinical reports, case reports, preclinical studies, reviews, and in vitro studies were grouped separately so that mechanistic and indirect findings were not assigned the same evidential weight as direct clinical evidence.

Methodological Appraisal and Synthesis

Randomized trials were considered using RoB 2 principles, and nonrandomized comparative studies were considered using ROBINS-I principles [10,11]. Because complete domain-level assessment records were not available, the manuscript reports a design-specific methodological appraisal rather than definitive formal risk-of-bias judgments. Case reports, preclinical studies, reviews, and in vitro studies were considered narratively. Meta-analysis was not undertaken because clinical populations, PRF preparations, endpoints, follow-up periods, and effect measures were not sufficiently compatible. Results were grouped by directness and outcome domain: perfusion/angiogenesis, flap or graft viability, wound healing, scar remodeling, and oncologic safety.

Study Selection and Data Handling

**Figure 1:**
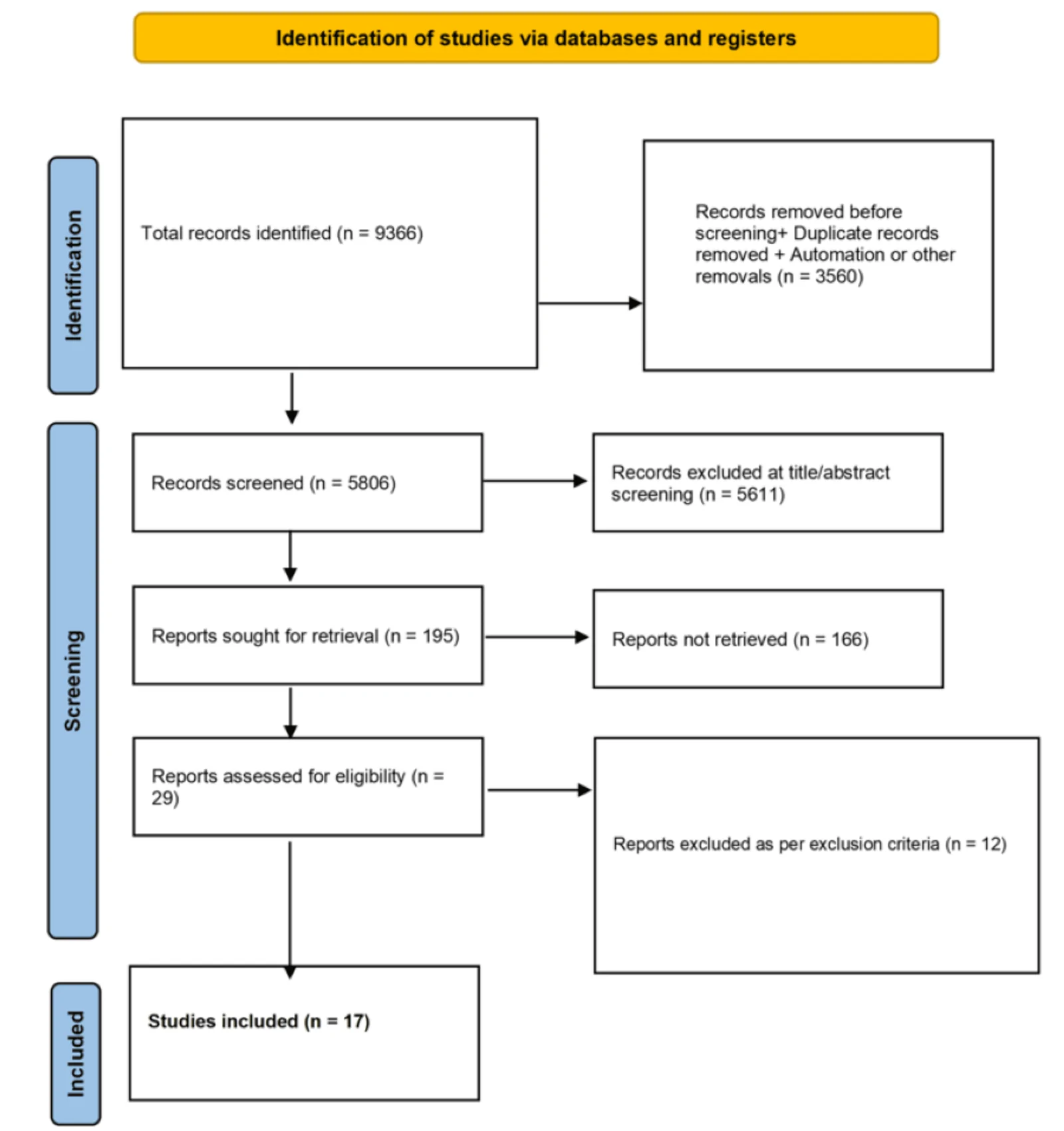
PRISMA flow chart.

The review used the documented information sources PubMed/MEDLINE, Scopus, Web of Science, the Cochrane Library, and reference-list searching. The available review file did not document prospective protocol registration, exact database search dates, a complete RIS/CSV export, duplicate handling, the number of screeners, disagreement resolution, or standardized data-extraction procedures (Figure 1).

Results

Study Selection and Evidence Base

Seventeen core reports were included in the qualitative synthesis: 16 human clinical reports and one preclinical flap study. Adjacent soft-tissue healing studies predominated; direct post-oncological facial flap studies were not identified. One prospective randomized study applied PRF directly to a reconstructive wound bed before skin grafting of the radial forearm flap donor site, a clinically relevant but non-equivalent context to local facial flap transfer [12]. One case report described repeated L-PRF use for delayed deep wound healing after tracheotomy or lymph-node dissection [13]. One preclinical perforator-flap model directly evaluated flap survival and vascularization [14]. The remaining core studies addressed scar remodeling, donor-site healing, postoperative swelling, epithelialization, MRONJ, and chronic wounds [15-28]. One in vitro oncologic safety study was retained only for contextual interpretation [8]. 

Characteristics of Included Studies

The included studies were heterogeneous in setting, tissue type, and PRF protocol. PRF was delivered as membranes, plugs, injectable PRF, liquid blood concentrate, or other specified derivatives. Follow-up ranged from the immediate postoperative period to several months; chronic-wound and facial-skin studies generally had longer clinically relevant follow-up than extraction or donor-site trials. Sample sizes were generally small, and objective imaging was uncommon. No study evaluated a powered cohort of post-oncological facial flap patients using standardized objective perfusion endpoints.

The characteristics of the included core reports are summarized in Table 1.

**Table 1:** Characteristics of included core reports. A-PRF, Advanced Platelet-Rich Fibrin; CGF, Concentrated Growth Factor; FGG, Free Gingival Graft; I-PRF, Injectable Platelet-Rich Fibrin; IHC, Immunohistochemistry; L-PRF, Leukocyte- and Platelet-Rich Fibrin; MRONJ, Medication-Related Osteonecrosis of the Jaw; OHIP-14, Oral Health Impact Profile-14; PRF, Platelet-Rich Fibrin; PRP, Platelet-Rich Plasma; RCT, Randomized Controlled Trial

Study	Country and design	Sample	Defect/tissue context	PRF protocol	Follow-up/outcomes
Straub et al. (2022) [12]	Germany; Prospective randomized clinical study	32	Radial forearm flap donor site	PRF membranes between wound bed and skin graft	Early graft healing/survival
Fourneau et al. (2023) [13]	Belgium; Case report	1	Delayed deep wound after tracheotomy/lymph-node dissection	Repeated L-PRF application	Long-term clinical healing
Li and Jin (2025) [14]	China; Animal perforator-flap model	Rats	Dorsal cross-region perforator flap	PRF vs PRP vs CGF vs control	Thermography, angiography, IHC, survival
Diab et al. (2023) [15]	Egypt; Comparative clinical study	30	Atrophic acne scars	Fluid PRF ± needling vs PRP	Scar grading/satisfaction
Majewska (2023) [16]	Poland; Retrospective facial skin study	10	Periorbital skin thinning/wrinkles	PRF injection protocol	High-frequency ultrasound
Majewska et al. (2024) [17]	Poland; Prospective open-label randomized study	20	Facial skin bioregeneration	PRP fractions and I/F-PRF from one draw	Ultrasound density/thickness
Majewska et al. (2025) [18]	Poland; Single-center ultrasound study	20	Facial skin density/thickness	PRP/PRF fractions	Ultrasound by age group
Gulsever and Uckan (2025) [19]	Turkey; Prospective randomized trial	Not reported in abstract	Palatal FGG donor wounds	L-PRF membrane dressing	Pain, bleeding, wound area, healing index
Scott et al. (2024) [20]	USA; Randomized controlled trial	Not reported in abstract	Palatal FGG donor site	PRF vs standard healing	Wound healing after graft harvest
Şen et al. (2024) [21]	Turkey; Randomized clinical trial	39	Palatal donor sites after FGG	L-PRF or A-PRF bandage	Pain, analgesics, OHIP-14, bleeding
Ceylan Şen et al. (2025) [22]	Turkey; Randomized clinical trial	67	Palatal donor sites	I-PRF vs hyaluronic acid vs control	Pain, epithelialization, color match
Bozkurt and Özdemir (2025) [23]	Turkey; Randomized controlled trial	36	Palatal wound after FGG	I-PRF vs photobiomodulation vs control	Wound area, pain, analgesia
Zwittnig et al. (2024) [24]	Austria; Split-mouth randomized trial	25 patients/100 teeth	Third molar flap/extraction wounds	Solid PRF clots	3D swelling, painkiller use, healing
Barone et al. (2025) [25]	Italy; Split-mouth randomized trial	32	Mandibular third molar sockets	PRF plug/membrane	3D facial swelling, pain, adverse events
Aliberti et al. (2025) [26]	Italy; Double-blind RCT	56	Impacted lower third molar extraction	Injectable PRF	Swelling, pain, wound healing
Nowak et al. (2026) [27]	Poland; Pilot clinical study	22	MRONJ surgical wounds	PRF adjunct vs no PRF	Pain, fistulas, soft-tissue healing, imaging
Lin et al. (2025) [28]	China; Retrospective cohort	Not reported in public abstract	Chronic wounds	PRF vs conventional wound care	Healing time, recurrence, scar formation

Outcomes of Tissue Perfusion and Angiogenesis

The principal limitation was the absence of direct human perfusion studies in post-oncological facial flaps. Straub et al. reported that PRF membranes placed between the wound bed and skin graft during radial forearm flap donor-site reconstruction were associated with higher graft survival (93.44% versus 86.96%; absolute difference, 6.48% points; p = 0.0292) [12]. A 95% confidence interval for the between-group difference was not available in the extracted data. This was not a facial flap perfusion study, but it is clinically relevant because graft take depends on plasmatic imbibition, inosculation, and revascularization. None of the included human studies used laser Doppler flowmetry, indocyanine green angiography, near-infrared spectroscopy, or tissue oxygenation monitoring to evaluate post-oncological facial flaps.

The rat dorsal cross-region perforator-flap model provided the clearest direct preclinical perfusion signal. Li and Jin reported that PRP, PRF, and concentrated growth factor promoted vascularization and flap survival, with PRF and concentrated growth factor showing stronger vascularization than PRP [14]. This supports biological plausibility but does not establish clinical benefit in irradiated facial beds or human post-oncological flap designs. Mechanistic reviews describe growth-factor release, leukocyte effects, fibrin-matrix functions, and wound-modulating properties that could support angiogenesis and tissue repair [4,5]. Conversely, in vitro work in nonmelanoma skin cancer cells showed changes in proliferation and cell-cycle- or apoptosis-related gene expression after exposure to liquid blood concentrates [8]. These findings are not evidence of clinical recurrence risk, but they support caution when oncologic clearance is uncertain. 

Perfusion and angiogenesis outcomes are summarized in Table 2.

**Table 2:** Perfusion and angiogenesis outcomes. L-PRF, Leukocyte- and Platelet-Rich Fibrin; CGF, Concentrated Growth Factor; NMSC, Nonmelanoma Skin Cancer; IHC, Immunohistochemistry; PRP, Platelet-Rich Plasma

Study	Model/context	Perfusion/angiogenesis measure	Key finding	Interpretation
Blanco et al. (2025) [4]	Mechanistic review	L-PRF molecular components	Describes anti-inflammatory, hemostatic, antimicrobial, soft-tissue regenerative pathways	Mechanistic rationale
Dohle et al. (2024) [8]	In vitro NMSC cells	Cell proliferation; cell-cycle/apoptosis-related gene expression	In vitro changes in proliferation and related gene-expression outcomes; not a healing-efficacy study	Oncologic safety caution
Straub et al. (2022) [12]	Human head/neck reconstructive donor site	Graft take as indirect vascularization endpoint	93.44% vs 86.96% graft survival; absolute difference 6.48 percentage points; p = 0.0292	Indirect support only
Li and Jin (2025) [14]	Rat perforator flap	Thermal imaging, angiography, IHC	PRF promoted vascularization and flap survival; PRF/CGF > PRP for vascularization	Preclinical support

No eligible clinical study directly evaluated partial necrosis, total necrosis, or flap loss after PRF-enhanced post-oncological facial local-flap reconstruction. The nearest clinical proxy was the radial forearm flap donor-site skin-graft study [12]. The preclinical perforator-flap model provided a signal of improved survival and vascularization [14], but animal dorsal flaps are not equivalent to human facial rotation, advancement, or transposition flaps. Accordingly, the evidence is insufficient rather than demonstrably unfavorable: no conclusion about routine PRF use for facial oncologic flap survival can be drawn.

Flap viability and survival outcomes are summarized in Table 3.

**Table 3:** Flap viability and survival outcomes. PRF, Platelet-Rich Fibrin

Setting	Evidence volume	Reported viability outcome	Conclusion
Clinical facial local flap after cancer excision	0 eligible studies	No partial/total necrosis data	No evidence for routine PRF use
Head/neck grafted donor site [12]	1 prospective randomized study	Improved graft survival	Relevant bed-conditioning proxy
Preclinical perforator flap [14]	1 animal study	Improved survival/vascularization	Biologic plausibility only

Wound Healing Outcomes

Adjacent clinical evidence was more informative for soft-tissue healing than for flap viability. Randomized trials at palatal donor sites reported improvements in selected outcomes, including pain, bleeding, wound area, healing indices, analgesic use, or patient-reported morbidity after free gingival graft harvesting [19-23]. Third-molar surgery trials reported reductions in selected measures of facial swelling, analgesic use, pain, or early wound-healing burden, although effects were not uniform across all endpoints [24-26]. In MRONJ, Nowak et al. evaluated PRF as a surgical adjunct using pain, fistula, soft-tissue healing, and imaging outcomes [27]. Lin et al. reported shorter healing time in a retrospective chronic-wound cohort treated with PRF and also assessed recurrence and scar formation [28].

Wound-healing outcomes are summarized in Table 4.

**Table 4:** Wound-healing outcomes. FGG, Free Gingival Graft; HA, Hyaluronic Acid; I-PRF, Injectable Platelet-Rich Fibrin; MRONJ, Medication-Related Osteonecrosis of the Jaw; PBMT, Photobiomodulation Therapy; QoL, Quality of Life; RCT, Randomized Controlled Trial

Study	Context	Main healing signal	Limitation
Gulsever and Uckan (2025) [19]	Palatal FGG donor wound	Lower pain/bleeding, reduced wound area, improved healing indices	Small RCT; oral mucosa
Şen et al. (2024) [21]	Palatal donor site	Improved morbidity/QoL and reduced analgesic use	Small RCT
Ceylan Şen et al. (2025) [22]	Palatal donor site	I-PRF improved discomfort and epithelialization vs control; HA superior overall	Comparative adjunct evidence
Bozkurt and Özdemir (2025) [23]	Palatal wound	I-PRF and PBMT accelerated healing and reduced pain/analgesia	Comparative RCT
Zwittnig et al. (2024) [24]	Third molar wounds	Less swelling at day 14 and lower painkiller use; other endpoints inconsistent	Split-mouth RCT
Barone et al. (2025) [25]	Third molar wounds	Reduced 3D facial swelling; no adverse reactions reported	Objective swelling imaging
Aliberti et al. (2025) [26]	Third molar wounds	Reduced swelling/pain and faster wound healing	Double-blind RCT
Nowak et al. (2026) [27]	MRONJ wounds	Pain and soft-tissue healing assessed	Pilot; oncologic-adjacent compromised tissue
Lin et al. (2025) [28]	Chronic wounds	Shorter healing time: 36.2 ± 9.3 vs 60.4 ± 11.4 days; absolute difference -24.2 days; p < 0.001	Retrospective; not facial/oncologic flap

Scar Remodeling Outcomes

Scar-remodeling evidence was indirect. Diab et al. reported improvement in atrophic acne scars with fluid PRF, alone or combined with needling, and greater response than PRP in the studied context [15]. Majewska reported ultrasound-assessed changes in periorbital skin density in a small retrospective study [16]. A subsequent prospective open-label randomized study developed a reproducible PRP/PRF preparation and ultrasound-imaging procedure for aesthetic treatment [17]. A 2025 single-center ultrasound study again reported increases in facial skin density and thickness across age groups, particularly in the lower-eyelid region [18]. These findings support a plausible remodeling effect but do not establish benefit for post-oncological facial scars; none of these studies used the Patient and Observer Scar Assessment Scale (POSAS) or the Vancouver Scar Scale (VSS) after facial oncologic reconstruction.

Scar-remodeling outcomes are summarized in Table 5.

**Table 5:** Scar remodeling outcomes.

Study	Tissue model	Outcome tool	Finding	Applicability
Diab et al. (2023) [15]	Atrophic acne scars	Scar grading/patient satisfaction	PRF response higher than PRP; mild side effects	Scar proxy, not surgical oncology
Majewska (2023) [16]	Periorbital skin	High-frequency ultrasound	Improved skin density in small cohort	No oncologic defects
Majewska et al. (2024) [17]	Facial bioregeneration	Ultrasound protocol	Reproducible preparation and ultrasound-assessment procedure	Aesthetic population
Majewska et al. (2025) [18]	Facial skin	Ultrasound density/thickness	Significant improvements, especially lower eyelid	Excluded skin cancer history

Methodological Appraisal and Certainty of Evidence

**Figure 2:**
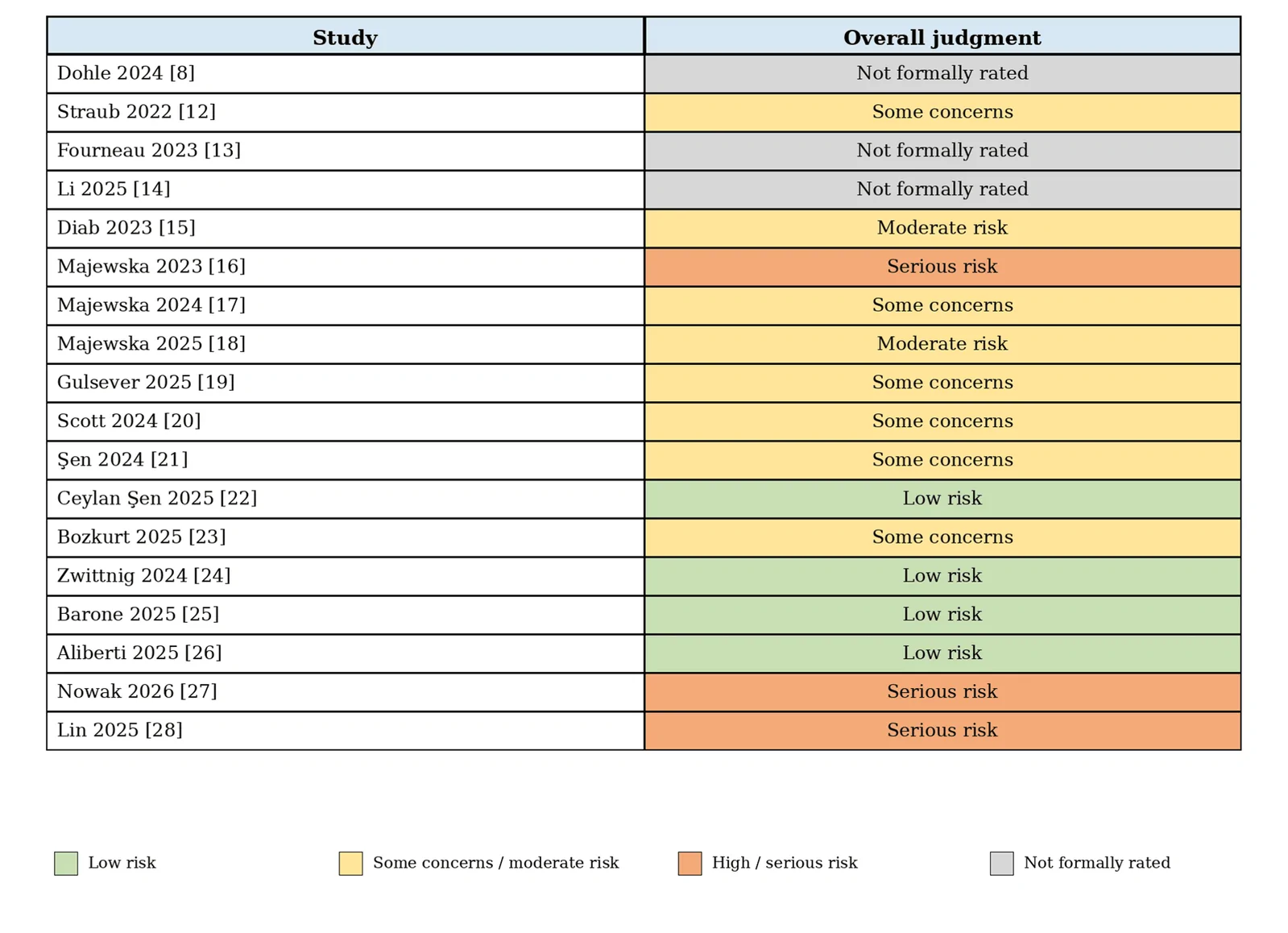
Risk-of-bias summary. Green = low risk; yellow = some concerns or moderate risk; orange = high or serious risk; grey = not formally rated with RoB 2 or ROBINS-I.

Design-specific methodological appraisal: Methodological quality was mixed. This was a design-specific methodological appraisal, not a complete domain-level risk-of-bias assessment. Randomized trials were considered using RoB 2 principles, and nonrandomized comparative studies were considered using ROBINS-I principles [10,11]. Because domain-level assessment records were not available, the overall judgments in Figure 2 and Table 6 should be interpreted as provisional. Case reports, animal studies, reviews, and in vitro studies were retained for contextual or mechanistic purposes but were not assigned formal RoB 2 or ROBINS-I judgments. Across the randomized oral and maxillofacial studies, recurring concerns included limited blinding, subjective pain or healing endpoints, small samples, and short follow-up. For the overall body of evidence, indirectness remained the dominant limitation because direct post-oncological facial flap studies with objective perfusion or validated scar outcomes were absent. An overview of the design-specific methodological appraisal is shown in Figure 2. The design-specific methodological appraisal is summarized in Table 6. 

**Table 6:** Design-specific methodological appraisal.

Study	Framework	Provisional overall concern	Principal limitation
Dohle et al. (2024) [8]	Narrative appraisal	Not formally rated	In vitro oncologic safety study
Straub et al. (2022) [12]	RoB 2	Some concerns	Randomization reported; indirect donor-site proxy and limited blinding detail
Fourneau et al. (2023) [13]	Narrative appraisal	Not formally rated	Single case report
Li and Jin (2025) [14]	Narrative appraisal	Not formally rated	Preclinical animal model
Diab et al. (2023) [15]	ROBINS-I	Moderate risk	Comparative scar study; indirect population and potential confounding
Majewska (2023) [16]	ROBINS-I	Serious risk	Very small retrospective study
Majewska et al. (2024) [17]	RoB 2	Some concerns	Open-label aesthetic study and indirect population
Majewska et al. (2025) [18]	ROBINS-I	Moderate risk	Single-center aesthetic population; indirectness
Gulsever and Uckan (2025) [19]	RoB 2	Some concerns	Small oral-mucosal donor-site trial
Scott et al. (2024) [20]	RoB 2	Some concerns	Subjective healing outcomes and limited blinding detail
Şen et al. (2024) [21]	RoB 2	Some concerns	Short follow-up and patient-reported outcomes
Ceylan Şen et al. (2025) [22]	RoB 2	Low risk	Randomized comparative design; indirect tissue context
Bozkurt and Özdemir (2025) [23]	RoB 2	Some concerns	Small randomized trial
Zwittnig et al. (2024) [24]	RoB 2	Low risk	Split-mouth randomized design; mixed endpoints
Barone et al. (2025) [25]	RoB 2	Low risk	Short-term swelling outcome but objective 3D assessment
Aliberti et al. (2025) [26]	RoB 2	Low risk	Randomized extraction model; indirect context
Nowak et al. (2026) [27]	ROBINS-I	Serious risk	Pilot clinical study with limited control of confounding
Lin et al. (2025) [28]	ROBINS-I	Serious risk	Retrospective chronic-wound cohort with confounding risk

The certainty of evidence is summarized in Table 7.

**Table 7:** Certainty-of-evidence assessment. RCT, Randomized Controlled Trial; POSAS, Patient and Observer Scar Assessment Scale; VSS, Vancouver Scar Scale; NMSC, Nonmelanoma Skin Cancer

Outcome	Evidence base	Key limitations	Certainty	Rationale
Tissue perfusion/angiogenesis	1 human proxy + 1 animal model	Serious risk of bias and inconsistency; very serious indirectness; publication bias not assessed	Very low	No human facial oncologic flap perfusion imaging
Flap/graft survival	1 human graft-bed study + 1 animal flap model	Serious risk of bias, inconsistency, and indirectness; publication bias not assessed	Very low	Signal exists for graft take, not facial flap survival
Wound healing/morbidity	Multiple small RCTs and cohorts	Some risk-of-bias concerns; serious inconsistency and indirectness; publication bias not assessed	Low	Consistent adjacent signal but indirect tissue context
Scar remodeling	Small aesthetic and scar studies	Serious risk of bias and inconsistency; very serious indirectness; publication bias not assessed	Very low	No POSAS/VSS evidence in oncologic facial flap scars
Oncologic safety	In vitro NMSC study + biologic reviews	Serious risk of bias, inconsistency, and indirectness; publication bias not assessed	Very low	Clinical safety studies are required after confirmed tumor clearance

Discussion

Principal Findings

The principal finding is that a direct clinical evidence base for PRF as a wound-bed conditioning adjunct in post-oncological facial flap reconstruction has not yet been established. The randomized radial forearm donor-site study is the strongest human reconstructive proxy and reported improved skin-graft survival when PRF was placed between the wound bed and graft [12]. The clearest perfusion signal is preclinical rather than clinical [14]. Oral and maxillofacial donor-site and extraction trials provide the most robust adjacent clinical evidence for selected wound-healing outcomes [19-26]. Evidence relevant to scar remodeling comes from acne-scar and facial-skin studies rather than oncologic flap scars [15-18].

Mechanistic Interpretation

PRF has a plausible mechanistic basis. The fibrin matrix provides a provisional scaffold and can support sustained local release of bioactive mediators. Reviews of L-PRF describe platelet- and leukocyte-mediated pathways relevant to inflammatory modulation, hemostasis, antimicrobial effects, soft-tissue regeneration, and growth-factor signaling [4]. Clinical guidance also emphasizes that PRF is not a uniform product: centrifugation method, clot age, membrane compression, tube chemistry, and liquid versus solid formulation can materially alter the final preparation [5]. Such heterogeneity may partly explain variable findings across adjacent clinical studies. Matrix stability, fibroblast migration, collagen organization, and angiogenic signaling are plausible mechanisms through which PRF could support a compromised wound bed; however, mechanistic plausibility does not establish improved human flap perfusion.

Evidence From Adjacent Clinical Contexts

Oral-surgery and maxillofacial literature has evaluated PRF more extensively than facial oncologic reconstruction. Narrative and systematic reviews describe applications in extraction sockets, oroantral communication, periodontal wounds, MRONJ, and chronic extraoral wounds, while also emphasizing heterogeneity and limited standardization [6,29-32]. MRONJ is anatomically and clinically distinct from facial skin-flap reconstruction, but it remains relevant as an example of a compromised surgical bed affected by antiresorptive or antiangiogenic therapies. Dermatologic evidence is also adjacent rather than direct: PRF may improve selected measures of skin texture, wrinkles, or scar appearance, but those findings cannot be transferred uncritically to patients with post-oncological facial scars, prior radiotherapy, altered lymphatics, or ongoing oncologic surveillance [33].

Clinical implications

At present, PRF should be regarded as an investigational adjunct rather than a proven standard for post-oncological facial flap reconstruction. Routine clinical use cannot be recommended outside a clearly documented research or protocol-based setting after confirmed oncologic clearance. Existing evidence is too indirect to justify recommending PRF for selected high-risk beds. Patients should not be told that PRF prevents flap necrosis, improves post-oncological facial scars, or has established oncologic safety. The most accurate clinical characterization is that PRF is biologically plausible and supported by adjacent wound-healing evidence but remains unproven for post-oncological facial flap perfusion, survival, and scar remodeling. 

Within a research or protocol-based setting, PRF must remain adjunctive to established reconstructive principles: confirmed tumor clearance, tension-free closure, adequately vascularized tissue, infection control, dead-space management, smoking cessation, nutritional optimization, and close postoperative monitoring. PRF cannot compensate for poor flap design, excessive tension, residual tumor, or an irradiated bed that requires more substantial vascularized tissue transfer.

Limitations

This review is limited by the scarcity of direct evidence, heterogeneity of PRF preparations, small samples, short follow-up, variable endpoints, and indirect tissue contexts. Objective human perfusion endpoints were largely absent, and post-oncological facial flap cohorts using validated scar instruments such as POSAS or the VSS were not identified. The restricted 2022-2026 English-language window may have excluded earlier or non-English evidence and limits historical comprehensiveness. In addition, prospective protocol registration, exact database search dates, database-specific strategies, duplicate handling, independent screening procedures, disagreement resolution, and standardized data-extraction methods were not documented in the available review file. A complete database export and deduplication log were unavailable for independent audit; consequently, database-level PRISMA counts were not presented. Effect estimates and 95% confidence intervals were also not consistently available in the extracted data. These limitations reduce confidence in the completeness and reproducibility of the evidence map. 

Future research

Further uncontrolled case series would add limited value. The priority is multicenter randomized or prospective comparative research using standardized PRF preparation, documented tumor clearance, stratification by radiotherapy and smoking status, blinded assessment, and follow-up of at least 6-12 months. A pragmatic comparator should be standard reconstruction without PRF. A preferred primary endpoint would combine a prespecified objective perfusion measure in the early postoperative period with partial or total flap necrosis by 30 days; validated scar outcomes should be secondary endpoints at 6-12 months. Sample-size calculations should be based on a clinically meaningful between-group difference in the primary endpoint, with allowance for loss to follow-up and center effects. Adoption should be protocol-driven and evidence-based rather than assumption-driven or marketing-led.

## Conclusions

PRF is a plausible autologous adjunct for reconstructive wound-bed conditioning. Evidence published from 2022 through 2026 in adjacent clinical contexts suggests potential benefits for skin-graft take, selected soft-tissue healing outcomes, postoperative swelling or pain, and dermal remodeling. Direct clinical evidence for improved perfusion, viability, or scar remodeling in post-oncological facial flaps, however, is lacking. This absence of direct evidence does not demonstrate that PRF is ineffective; it demonstrates that routine use for this indication is not evidence-based. PRF should be evaluated in standardized oncologic reconstructive trials with objective perfusion, viability, and validated scar outcomes before routine clinical adoption.
